# Association between perceived harm of tobacco and intention to quit: a cross-sectional analysis of the Vietnam Global Adult Tobacco Survey

**DOI:** 10.1186/s12889-022-13348-w

**Published:** 2022-05-06

**Authors:** Thi Phuong Thao Tran, Jinju Park, Thi Ngoc Phuong Nguyen, Van Minh Hoang, Min Kyung Lim

**Affiliations:** 1 Department of Cancer Control and Population Health, Graduate School of Cancer Science and Policy, National Cancer Center, 323 Ilsan-ro Ilsandong-gu, Goyang-si, Gyeounggi-do 410-769 Republic of Korea; 2 Center for Population Health Sciences, Hanoi University of Public Health, Hanoi, Vietnam; 3 College of Medicine, Inha University, 100 Inha-ro, Michuhol-gu, Incheon, 22212 Republic of Korea

**Keywords:** Bamboo waterpipe tobacco, Intention to quit, Perceived harm, Vietnam

## Abstract

**Background:**

Perception of harm plays an important role in predicting intention to quit—an integral part of the cessation process. Perception on harm from bamboo waterpipe tobacco was unknown, even the predominant of this type of tobacco use. This study investigated the effects of perceived harm from cigarette and bamboo waterpipe tobacco on intention to quit among adult male Vietnamese tobacco users.

**Methods:**

From the nationally-representative 2015 Global Adult Tobacco Survey, we included 1,351 adult males (≥ 18 years old) who used cigarettes, bamboo waterpipes, or both. Demographic characteristics, tobacco use behaviors, perceived harm from tobacco use, and regulation/policy exposure were measured. Effects of perceived harm from cigarette and bamboo waterpipe tobacco on intention to quit were assessed by logistic regression.

**Results:**

Intention to quit prevalence was 59.0%, 55.0%, and 58.4% for cigarette, waterpipe, and dual users, respectively. Tobacco users who perceived that “using cigarettes and/or waterpipe could cause severe illness” and “waterpipe use is less harmful than cigarette smoking”, had tobacco use bans at home, or were exposed to anti-smoking campaigns or encouragement to quit information were more likely to intend to quit. When analyzed by tobacco users, intention to quit was more likely for those users who perceived their tobacco product as more harmful than the other product type, although statistical significance was only observed for cigarette users.

**Conclusions:**

Misperceptions regarding harm from tobacco use could negatively affect intention to quit. Dissemination of accurate information on the risks from all forms of tobacco use and enforcement of tobacco control policies are important for encouraging intention to quit.

**Supplementary Information:**

The online version contains supplementary material available at 10.1186/s12889-022-13348-w.

## Introduction

Despite the predominant form of cigarettes among various tobacco types all over the world, waterpipe use also significantly contributed to the growing share of tobacco use globally. The global prevalence of waterpipe use that ranges from 5 to 34%, with higher estimates in rural Western Pacific and Eastern Mediterranean regions and increasing use among youths and adolescents in European countries [1]. Although bamboo waterpipe use common in the Western Pacific region, including Vietnam, research on the effects of its use has not been yet received attention properly [1]. In Vietnam, the prevalence of smoking is high, with nearly half of men identified as current tobacco users [2]. Among Vietnamese men, the prevalence of cigarette smoking is 36.7%, and the prevalence of bamboo waterpipe use is 13.7% [2], which is the highest prevalence of waterpipe use in Asia [1]. Nevertheless, there is no study investigated on bamboo waterpipe user in Vietnam.

Given the concept of the theory of planed behavior with respect to smoking [3], the intention to quit smoking forms an integral part of the cessation process and has been found to be significantly associated with quit attempt [4, 5]. However, a study of smokers from 14 low-and-middle-income countries indicated that only 18% of smokers plan to quit smoking [6], whereas the rate is 2 to 3 times higher in many high-income countries with well implemented tobacco control policies [7, 8]. Thus, investigation of factors associated with a lower prevalence of intention to quit, especially in LMICs, is crucial to the development of effective tobacco control policies. Several factors related to intention to quit have been previously investigated, including demographics [6, 7, 9, 10], tobacco-related knowledge [11–13], risk perception [14–16], socio-contextual [17], and regulation/policy effects [6, 7, 15, 18]. Among these associated factors, perceived risk plays an important role in predicting health behaviors as hypothesized in the Health Belief Model [19]. The tobacco-related studies have been supported the conceptual model that harm from tobacco product was significantly predicted intention to quit and quit attempt [20]. However, almost study was focus on conventional cigarette or emerging tobacco product such as e-cigarette or heated tobacco products, while no study investigated effects of perception on comparative harm from bamboo waterpipe tobacco and cigarette on intention to quit has been conducted, even the predominant of this type of tobacco use.

In Vietnam, a few studies have investigated factors associated with smoking cessation patterns among Vietnamese tobacco users including age [21, 22], living area [22], level of nicotine dependence [21, 23], past quit attempts [21], pictorial health warning [24], knowledge of illness caused by smoking [22]. However, studies that assess the impact of perceived harm from different tobacco product types including waterpipe tobacco on intention to quit and that take individual and policy factors into consideration has not investigated yet. Hence, aim of this study was to investigate the effect of perceived harm from cigarette and bamboo waterpipe tobacco on the intention to quit among a nationally representative sample of male tobacco users in Vietnam.

## Methods

### Data source and study population

Data were obtained from the 2015 Global Adult Tobacco Survey (GATS) in Vietnam, which is a cross-sectional nationally representative survey of 8,996 Vietnamese participants who were ≥ 15 years old. Standardized approaches for sampling method, questionnaire design, data collection, data management, and ethical considerations were used for conducting GATS. The questionnaire addressed 10 sections related to the World Health Organization’s MPOWER measures to assist countries with tobacco control: (1) demographic characteristics, (2) tobacco smoking, (3) electronic cigarette use, (4) smokeless tobacco use, (5) cessation efforts, (6) secondhand smoke exposure, (7) economics, (8) media exposure, (9) knowledge, attitudes and perceptions, and (10) pictorial graphic health warnings and tax stamps on cigarette packs.

**Fig. 1:**
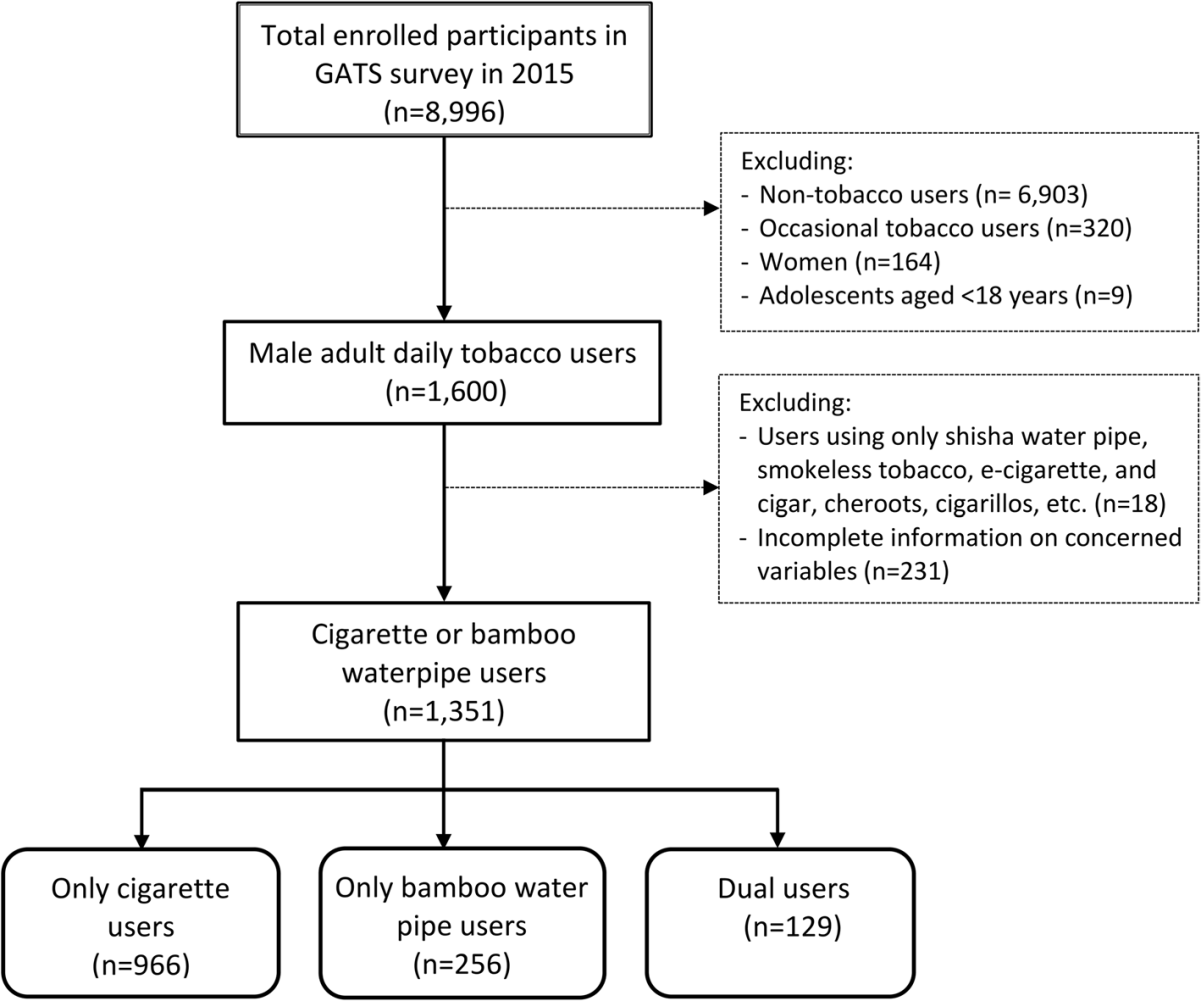
Flowchart for selection of study participants

Tobacco users were defined as who reported that they currently smoke any kinds of tobacco (e.g., cigarette, bamboo waterpipe, smokeless tobacco, etc.) on a daily basis or less than daily. After excluding non-tobacco users and occasional tobacco users, women, and those < 18 years old, 1,600 adult male tobacco users were available for inclusion in this study. We excluded the non-daily tobacco users because of lacking several information on smoking behaviors such as first use of a cigarette/waterpipe after waking among non-daily smokers, which was a well-known factor (i.e., nicotine dependence) associated with the intention to quit. After excluding 18 tobacco users who used these other minor types of tobacco products (shisha, smokeless tobacco, e-cigarettes and cigars) and 231 users who had incomplete information on concerned variables, 1,351 tobacco users were included in the final analysis, including 966 users of cigarettes only, 256 users of bamboo waterpipes only, and 129 dual users (Fig. 1).

### Description of variables

The main outcome of this study is intention to quit. All tobacco users who planned to quit “within the next month”, “within the next 12 months”, or “someday but not in the next 12 months” were classified as having the intention to quit. Those who answered “not interested in quitting” were classified as not having the intention to quit, which was also defined in previous study [25].

The perceived harm from tobacco use were independent variable, including knowledge of whether cigarette and waterpipe use causes serious illness (both do not cause severe illness, only waterpipe causes severe illness, only cigarette causes severe illness, or both products cause severe illness), and perceived harm from waterpipe use versus cigarette smoking (less harmful, equally harmful, or more harmful).

**Fig. 2:**
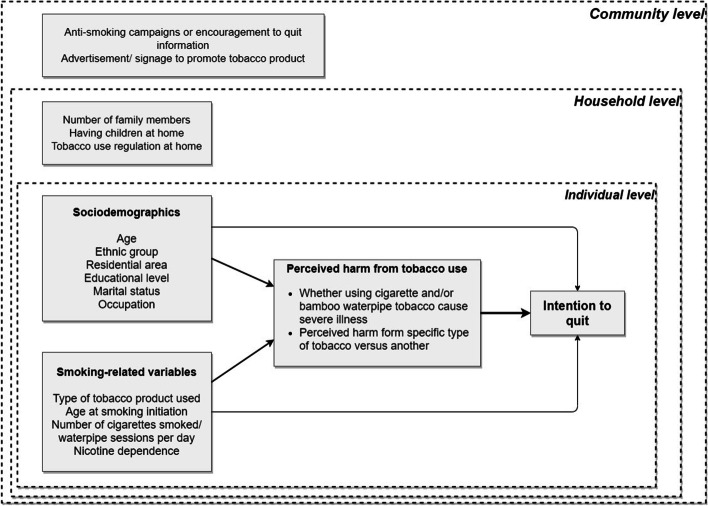
Conceptual diagram on the factors associated with intention to quit

For covariates, the factors groups associated with intention to quit were illustrated in conceptual diagram in Fig. 2. For individual level, demographic characteristics included age (18–24, 25–44, 45–65, or ≥ 65 years), ethnic group (Kinh-major ethnicity, or others—minor ethnicity such as Thai, Tay, Nung, Dao, etc.), residential area (rural or urban), education level (primary school or less, secondary school, high school or higher), marital status (unmarried; married; or separated, divorced or widowed). Occupation was classified as professionals or managers (e.g., legislators, senior officials, or managers; high qualified professionals; or technicians or associate professionals), skilled laborers (e.g., members of armed forces; service workers; shop and market sales workers; skilled agricultural and fishery workers; craft and related trade workers; or plant and machine operators and assemblers), semi-skilled laborers or clerks (e.g., elementary occupation, clerks, drivers, or guardians), and others (e.g., student, homemaker, retired, or unemployed).

Information on tobacco use behaviors, including type of tobacco products used (cigarettes, waterpipe, or dual user) and age at tobacco use initiation were obtained. Among daily tobacco users, the number of cigarettes smoked and waterpipe sessions per day were asked for cigarette smokers and waterpipe users, respectively. Time to the first use of cigarette or waterpipe tobacco after waking (≤ 5, 6–30, 31–60, or > 60 min) was determined.

For household level, information on the number of family members, having children at home (yes or no), and tobacco use regulation at home (no ban, partial ban, or comprehensive ban) was obtained. No ban on smoking in household was defined as smoking is allowed in every room inside of home or there are no rules on smoking ban. Partial ban was defined as smoking is allowed in some rooms inside of home or smoking is generally not allowed inside of your home but there are exceptions. Smoking is never allowed inside of the home was comprehensive ban.

For community factors, tobacco control policy also was measured. Data were also recorded regarding whether exposure to pictorial health warnings on cigarette packs, anti-smoking campaigns or encouragement to quit information, and advertisements or signage to promote tobacco products within the last 30 days in locations such as newspapers, television, radio, or internet.

### Statistical analysis

The frequency distribution for each variable by intention to quit was performed and the collinearity of variables was evaluated. Multiple logistic regression analysis was used to evaluate the association between intention to quit and perceived harm on cigarette and bamboo waterpipe tobacco. The final model was selected after consideration of collinearity of variables of individual-level factors, adjustment for potential confounders including individual-level factors (age group, educational level, marital status), age at smoking initiation, number of cigarette smoked/waterpipe sessions used per day, time to the first use of cigarette or waterpipe tobacco after waking), household-level factors (smoking ban at home, having children at home), and community-level factors (Exposed to anti-smoking campaigns or encouragement to quit information, and exposed to advertisements/signage to promote tobacco products in the last 30 days), and assessment of model fit. Because of lacking the standardized measurement of intensity for both cigarette and waterpipe smoking, the number of cigarettes smoked and a number of waterpipe sessions used daily for cigarette users and waterpipe users was measured, respectively. Therefore, we combined two such variables into a single one to adjust in multiple logistic regression model among the whole study population. Subgroup analysis stratified by tobacco users was performed, and the reference group of a variable on perceived harm from waterpipe use versus cigarette smoking was changed. For cigarette-only users, perceived harm from cigarette smoking compared with waterpipe tobacco use was asked; in contrast, for waterpipe-only users, perceived harm from waterpipe use compared with cigarette smoking was measured. To examine the selection bias due to excluding 231 observations having missing information on concerning variables, we did the sensitivity analysis of factors associated with intention to quit by tobacco user groups, shown in Supplemental Table 1. Both descriptive and analytical statistical approaches were applied using weights. All statistical analyses were performed with STATA (version 14.0) software, and values of *p* < 0.05 were considered statistically significant.

**Table 1:** Factors associated with intention to quit: logistic regression analysis

	**Total**^**a**^	**Having intention**^**b**^	**Model 1**^**c**^		**Model 2**^**d**^	
	Weighted %	Weighted %	OR	(95%CI)	OR	(95%CI)
**Demographic characteristics**						
Age group						
18–24	11	65.2	1		1	
25–44	50.6	57.8	0.73	[0.42–1.28]	0.57	[0.31–1.06]
45–64	32.5	56.7	0.7	[0.41–1.19]	0.59	[0.30–1.15]
≥ 65	5.9	53.3	0.61	[0.32–1.15]	0.49	[0.23–1.04]
Ethnic group						
Kinh group	87.2	58.6	1			-
Others	12.8	55.6	0.88	[0.58–1.36]		-
Residential area						
Rural	68.1	58.1	1			-
Urban	31.9	58.3	1.01	[0.77–1.32]		-
Education level						-
Primary or less	16.3	51.6	1		1	
Secondary school	56.8	58	1.3	[0.90–1.86]	1.05	[0.72–1.53]
High school or higher	26.9	62.7	1.58*	[1.04–2.40]	1.16	[0.75–1.80]
Marital status						
Unmarried	16.6	61.2	1		1	
Married	80.4	57.5	0.86	[0.55–1.34]	0.97	[0.55–1.71]
Separated/divorced/widowed	3	59.7	0.94	[0.49–1.81]	1.34	[0.62–2.93]
Occupation						
Professionals/managers	5.8	74.3	1			-
Skilled labor	22.3	57.8	0.47*	[0.25–0.91]		-
Semi-skilled labor/clerks	62.5	57.2	0.46*	[0.25–0.86]		-
Others	9.4	56.6	0.45*	[0.22–0.93]		-
Number of family members, mean (ci)	4.1 (3.9–4.2)	4.1 (4.0–4.3)	1.04	[0.95–1.14]		-
Having children at home						
No	40.2	54.7	1		1	
Yes	59.8	60.5	1.27	[0.94–1.70]	1.36	[0.98–1.89]
**Tobacco use behaviors**						
Type of tobacco products used						
Cigarettes-only users	70.5	59.0	1		1	
Waterpipe tobacco-only users	19.1	55.0	0.85	[0.61–1.17]	0.75	[0.51–1.10]
Dual-users	10.4	58.4	0.97	[0.61–1.56]	0.97	[0.58–1.63]
Age at tobacco use initiation, mean (ci)	19.8 (19.4–20.1)	20.3 (19.8–20.7)	1.04***	[1.02–1.07]	1.05***	[1.02–1.07]
Number of cigarettes smoked/ waterpipe sessions per day, mean (ci)	15.3 (14.7–15.9)	14.9 (14.1–15.7)	0.99	[0.98–1.00]	1.00	[0.98–1.02]
Time to the first use of cigarette/ waterpipe after waking						
Within 5 min	17.9	53.4	1		1	
6–30 min	45.2	55.4	1.08	[0.76–1.54]	1.09	[0.75–1.58]
31–60 min	18.8	59.4	1.28	[0.85–1.91]	1.17	[0.75–1.83]
> 60 min	18.1	68.6	1.90**	[1.26–2.89]	1.62*	[1.00–2.63]
**Perceived harm from tobacco use**						
Cigarette and waterpipe use causes severe illness						
Do not cause severe illness	0.8	8.8	1		1	
Only waterpipe causes severe illness	2	44.4	8.32*	[1.43–48.21]	6.77*	[1.08–42.55]
Only cigarette causes severe illness	5.1	59.5	15.30**	[2.88–81.28]	10.56**	[2.00–55.83]
Both products cause severe illness	92.1	58.8	14.90***	[3.16–70.39]	9.58**	[2.03–45.24]
Perceived harm from waterpipe use versus cigarette smoking						
Less harmful	44.9	59.9	1		1	
Equally harmful	29.8	56.8	0.88	[0.64–1.20]	0.75	[0.52–1.07]
More harmful	25.3	56.8	0.88	[0.60–1.29]	0.72	[0.46–1.13]
**Regulation/policy effect**						
Tobacco use regulation at home						
No ban	60.5	53.3	1		1	
Partial ban	28.3	64.1	1.57**	[1.17–2.09]	1.52**	[1.12–2.04]
Comprehensive ban	11.2	70	2.04**	[1.31–3.20]	1.74*	[1.10–2.74]
Exposured to anti-smoking campaigns or encouragement to quit information (within the last 30 days)						
No	22.2	47.4	1		1	
Yes	77.8	61.3	1.76**	[1.25–2.48]	1.84***	[1.29–2.62]
Exposed to advertisements/signage to promote tobacco products (within the last 30 days)						
No	86.8	57.6	1		1	
Yes	13.2	62.3	1.22	[0.85–1.76]	1.07	[0.73–1.58]

^*^p < 0.05, ** *p* < 0.01, *** *p* < 0.001

^a^ Column proportions

^b^ Row proportions

^c^ Univariate model

^d^ Multiple model adjusted for all factors in the model, *n* = 1348

## Results

There were 1,351 tobacco users included in our study and majority aged from 25 to 64 years, was Kinh ethnicity, and lived in rural area (Table 1). 70.5% of them were cigarette-only smokers. Mean age at smoking initiation was 19.8. Nearly half of tobacco users perceived that waterpipe use was less harmful than cigarette smoking. 39.5% of households had a comprehensive or partial ban on tobacco use at home. Most tobacco users were exposed to pictorial health warnings on cigarette packs and anti-smoking campaigns or encouragement to quit information within the last 30 days.

Overall, 58.2% of tobacco users had the intention to quit (59.0%, 55.0%, and 58.4% of cigarette users, waterpipe users, and dual users, respectively). According to the multiple model, tobacco users were more likely to intend to quit smoking if they initiated smoking at older age, had higher nicotine dependence, perceived that severe illness could be caused by cigarette use only, waterpipe tobacco only or both waterpipe and cigarettes, had a partial or comprehensive tobacco ban at home, or were exposed to anti-smoking campaigns or encouragement to quit information within the last 30 days (Table 1).

The main findings of this study was shown in the subgroup analysis by type of tobacco product used (Table 2). In multiple model, for cigarette users, those who perceived that use of both cigarette and waterpipe tobacco causes severe illness had a higher likelihood of intention to quit than those thought that both did not cause severe illness. Perceiving that cigarette and waterpipe tobacco caused severe illness were also the associated factor of intention to quit among cigarette-only users and waterpipe tobacco-only users. Notably, 81.0% of waterpipe users perceived that waterpipe use was less harmful than cigarette smoking, whereas only 35.0% of cigarette users perceived that cigarette smoke was less harmful than waterpipe use. Furthermore, cigarette smokers who perceived their tobacco product of choice as more harmful than waterpipe tobacco smoking were more likely to have intention to quit than those that perceived their product as less harmful; however, a significant association was not observed for waterpipe users. Furthermore, having a smoking ban at home was significantly associated with a higher likelihood of intention to quit for users of waterpipe only; this association was not significant for cigarette-only users and dual users. Exposure to anti-smoking campaigns or encouragement to quit information within the last 30 days significantly increased the likelihood of intention to quit for dual users. Additionally, age at smoking initiation and time to the first use of cigarette/ waterpipe tobacco were significantly associated with intention to quit among cigarette-only users.

**Table 2:** Factors associated with intention to quit by tobacco user groups: multiple logistic regression analysis

	**Cigarette-only users**			**Waterpipe tobacco only-users**			**Dual users**		
	**Total**^**a**^	**Having intention**^**b**^	**Multiple model**^**c**^	**Total**^**a**^	**Having intention**^**b**^	**Multiple model**^**c**^	**Total**^**a**^	**Having intention**^**b**^	**Multiple model**^**c**^
	Weighted %	Weighted %	OR (95%CI)	Weighted %	Weighted %	OR (95%CI)	Weighted %	Weighted %	OR (95%CI)
**Demographic characteristics**									
Age group									
18–24	11.3	69.7	1	5.9	37.4	1	18	63.4	1
25–44	51.9	57.0	0.52 [0.24–1.10]	45.2	57	0.91 [0.17–4.96]	51.6	64.9	0.72 [0.08–6.52]
45–64	30.2	59.4	0.62 [0.27–1.38]	42.9	54.6	1.16 [0.19–6.95]	28.9	44.1	0.27 [0.02–3.04]
≥ 65	6.5	51.9	0.45 [0.18–1.11]	6.0	59.7	1.20 [0.15–9.82]	1.5	47.0	-
Education level									
Primary or less	19.4	51.4	1	11.2	47.5	1	4.5	74.2	1
Secondary school	54.1	60.5	1.12 [0.74–1.69]	66.5	49.8	0.83 [0.27–2.49]	57.2	59.4	0.24 [0.04–1.63]
High school or higher	26.5	61.6	1.16 [0.70–1.94]	22.3	74.4	2.03 [0.58–7.08]	38.3	55.0	0.16 [0.02–1.04]
Marital status									
Unmarried	18.7	64.0	1	6.7	37.5	1	20.4	57.9	1
Married	77.9	57.8	0.90 [0.46–1.76]	92.2	56.4	1 [0.17–5.98]	76.2	58.2	1.38 [0.17–11.00]
Separated/divorced/widowed	3.4	60.2	1.21 [0.50–2.92]	1.1	45.9	1.26 [0.12–12.95]	3.4	65.2	4.91 [0.31–77.02]
Having children at home									
No	40.6	57.4	1	41.7	45.6	1	34.6	53.6	1
Yes	59.4	60.2	1.28 [0.87–1.89]	58.3	61.7	1.8 [0.84–3.83]	65.4	60.9	1.04 [0.29–3.75]
**Tobacco use behaviors**									
Age at tobacco use initiation, mean (ci)	19.8 (19.4–20.1)	20.2 (19.7–20.6)	1.05* [1.01–1.08]	21.4 (20.4–22.5)	22.5 (21.0–24.0)	1.04 [1.00–1.09]	16.9 (16.3–17.5)	17.2 (16.3–18.2)	1.07 [0.95–1.20]
Number of cigarettes per day, mean (ci)	14.1 (13.5–14.7)	13.5 (12.8–14.2)	0.99 [0.97–1.02]	-	-	-	12.4 (10.9–14.0)	11.4 (9.3–13.4)	0.96 [0.88–1.04]
Number of WP sessions smoked per day, mean (ci)	-	-	-	14.3 (12.6–15.9)	14.6 (12.1–17.0)	1.01 [0.98–1.05]	12.5 (10.4–14.5)	13.2 (10.6–15.9)	1.02 [0.96–1.09]
Time to the first use of cigarette/waterpipe after waking									
Within 5 min	12.2	47.1	1	28.1	57.9	1	37.8	61.3	1
6–30 min	46	55.5	1.33 [0.83–2.12]	42.5	51.8	1.21 [0.61–2.41]	44.8	61.0	1.20 [0.30–4.73]
31–60 min	20.1	63	1.69 [0.98–2.89]	20	51.9	1.2 [0.47–3.03]	7.8	33.0	0.21 [0.04–1.30]
> 60 min	21.7	69.6	2.12** [1.21–3.70]	9.4	67.5	1.84 [0.48–7.05]	9.6	55.0	0.89 [0.13–5.97]
**Perceived harm from tobacco use**									
Cigarette and waterpipe use causes severe illness									
Do not cause severe illness	0.8	11.2	1	0.4	0	-	0.8	0	-
Only waterpipe causes severe illness	2.1	50.5	10.21* [1.49–69.80]	2.6	35.1	0.16* [0.03–0.83]	1	0	-
Only cigarette causes severe illness	5.6	68.1	13.54** [2.29–80.16]	2.5	53.1	1.87 [0.31–11.45]	6.2	10.4	0.07* [0.01–0.95]
Both cause severe illness	91.5	59.1	8.95** [1.81–44.29]	94.6	55.8	1	92.1	62.7	1
Perceived harm from their tobacco product compares to another									
Less harmful	30.3	56.1	1	81	53.1	1	-	-	-
Equally harmful	34.7	54.9	0.94 [0.62–1.44]	16.7	61.6	1.28 [0.50–3.28]	-	-	-
More harmful	35	67.2	1.72* [1.07–2.78]	2.2	73.6	1.55 [0.27–8.83]	-	-	-
**Regulation/policy effect**									
Tobacco use regulation at home									
No ban	59.5	56.1	1	61.6	42.1	1	64.7	55.2	1
Partial ban	29.3	59.9	1.1 [0.78–1.54]	27.2	77.6	4.42*** [1.98–9.85]	24.1	70.1	1.80 [0.50–6.42]
Comprehensive ban	11.2	72.5	1.58 [0.91–2.75]	11.2	70.9	3.05 [0.94–9.85]	11.2	51.1	1.11 [0.27–4.62]
Exposed to anti-smoking campaigns or encouragement to quit information (within the last 30 days)									
No	22.3	52.4	1	21.2	36.9	1	23	32.1	1
Yes	77.7	60.9	1.51 [1.00–2.29]	78.8	59.8	2 [0.78–5.12]	77	66.2	5.35* [1.49–19.26]
Exposed to advertisements/signage to promote tobacco products (within the last 30 days)									
No	86.2	58.9	1	90.9	53.9	1	83.4	55.7	1
Yes	13.8	60	1.07 [0.69–1.65]	9.1	66.1	1 [0.33–3.00]	16.6	71.5	2.04 [0.47–8.89]
N	963			255			125		

^*^p < 0.05, ** *p* < 0.01, *** *p* < 0.001

^a^ Column proportions

^b^ Row proportions

^c^Adjusted for all factors in the model

Supplemental Table 1 shows sensitivity analysis that examined factors associated with intention to quit by tobacco user groups. The association between the harm from tobacco products and intention to quit was not different from the main findings.

## Discussion

In the present study, Vietnamese waterpipe users had a lower likelihood of intention to quit than did cigarette smokers. In a previous study in Egypt, cigarette and mixed users had a significantly higher likelihood of intention to quit than did waterpipe (i.e., shisha) users [26]. Another previous study indicated that waterpipe use might be associated with the inducement of cigarette smoking relapse [27].

The use of the Vietnamese waterpipe has a long history in Vietnam, dating back to the eighteenth century. Its use has become a traditional custom of cultural and spiritual importance among Vietnamese men, even among women in ethnic minorities [28]. Furthermore, the lack of waterpipe-specific control policies in Vietnam, such as no tax on waterpipe tobacco, and no health warning, may explain the low prevalence of intention to quit among waterpipe users, especially given the social and culture acceptability of this product, which is similar what is seen in China [29], Egypt [26], and the US [30]. Thus, waterpipe-specific control policies and activities should be enacted and enforced such as prevention campaigns, warnings of harm associated with use, offering cessation programs, and raising taxation, in a manner similar to that for cigarette control policies that have been implemented in Vietnam with remarkable success [31]. Such efforts should aim to reduce the social acceptability of waterpipe use and encourage intention to quit among waterpipe users in Vietnam, where the prevalence of tobacco use is high but the past quit attempt and 6-month prolonged abstinence rates are still low at 39.6% and 5.1%, respectively [2, 32]. In addition, further studies are needed to identify effective smoking interventions and factors that contribute to the lower prevalence of intention to quit among waterpipe users.

In the present study, misunderstanding of the harm from tobacco products were identified as important independent factors associated with intention to quit among all tobacco users combined. The prevalence of intent to quit was lowest among tobacco users who perceived that waterpipe use was more harmful than cigarette smoking, of which most were cigarette smokers (188/195 users, 96.4%) (Data not shown). In other words, a considerable proportion of cigarette users, who are the predominant group of tobacco users, may be more likely to quit because they perceived that cigarettes were more harmful than the Vietnamese waterpipe. This finding was also observed in the subgroup analysis by product type, with cigarette smokers and waterpipe users who perceived their tobacco product of choice as more harmful than the other product type being more likely to have intention to quit than those that perceived their product as less harmful (Table 2).

It is notable that only 35.0% of cigarette users perceived that cigarette smoking was less harmful than waterpipe use, but most waterpipe users (81.0%) perceived that waterpipe use was less harmful than cigarette smoking, which is consistent with a significantly lower prevalence of intention to quit among waterpipe users. Our findings are relevant to marketing promotions of the tobacco industry for products such as the waterpipe (e.g., hookah), e-cigarettes, and heated tobacco as less harmful alternatives for cigarettes and as devices for smoking cessation [33–36]. However, evidence suggests that these alternative products can serve as a bridge to cigarette smoking [1, 37–42] and hinder attempts to quit and successful cessation among tobacco users [1, 27, 43]. Thus, to prevent the transition to alternative smoking products rather than quitting, widespread educational campaigns that counter marketing activities by tobacco companies are needed. In addition, marketing activities by tobacco companies should be regulated to prevent misperceptions regarding the harm from different smoking products. The present study also indicated that tobacco users who perceived that both cigarette and waterpipe use can cause severe illness were significantly more likely to have intention to quit than did those who did not perceive these products as harmful. This finding is consistent with previous studies that identify knowledge/perception about the harmful effects of tobacco products as factors that are strongly associated with the intention to quit [11–13]. However, in subgroup analysis by tobacco users, perceived harm from their tobacco product compares to another and perceived harm of whether cigarette and waterpipe use causes serious illness were not significantly associated with intention to quit among waterpipe users or dual users, which was likely the result of small sample size.

Additionally, we found that implementation of a comprehensive or partial ban on smoking at home was positively associated with intention to quit, which is consistent with findings in other countries [6, 7, 15]. Also similar to previous studies [6, 44], we found that likelihood of intention to quit was increased by exposure to anti-smoking campaigns or encouragement to quit information In subgroup analysis by tobacco product types, the significant association between smoking ban at home and intention to quit only showed in waterpipe-only users, not in cigarette-only smokers. The one of possible explanation is that waterpipe session duration usually lasts longer, even over one hour, than cigarette smoking [45, 46], it could thereby not be permitted to use waterpipe tobacco at home. Therefore, ban on smoking at home due to longer duration of waterpipe tobacco use could motivate intent to quit.

The present study highlighted that type of tobacco product and perceived harm from these products might be key factors affecting intention to quit among male tobacco users in Vietnam, where the quit attempt rate is low, and enforcement of tobacco control policies is lacking. However, a few limitations should be mentioned. First, given the cross-sectional study design, the temporal association between independent variables and intention to quit could not be established. However, our findings are meaningful and generalizable, given the use of a nationally representative sample of the Vietnamese population and standardized methods to conduct the survey [2]. Secondly, small sample sizes could explain why statistically significant associations between several potential factors and intention to quit were not observed for waterpipe and dual users, even though significant results were obtained for cigarette smokers. Third, the patterns and intensity of smoking for cigarette and waterpipe sessions are contrasting, however, it has not been available for the standardized measurement scale for both tobacco types. Therefore, we put number of cigarettes smoked and number of waterpipe session in the same categories for analysis for all smokers (Table 1). Lastly, users of other tobacco products, such as smokeless tobacco and e-cigarettes, were excluded from the present study because of the low prevalence of their use in Vietnam. Nevertheless, we do not believe that these exclusions had a meaningful effect on our results.

The promotion of certain tobacco product types as less harmful products could negatively impact intention to quit. Conversely, bans on tobacco use at home and exposure to anti-smoking campaigns or encouragement to quit information could increase the prevalence of intention to quit. Therefore, dissemination of accurate information on the health risks from all forms of tobacco and enforcement of tobacco control policies are important for encouraging intention to quit. Furthermore, those strategies might be applicable for recent emerging novel tobacco products, such as e-cigarettes and heated tobacco, which have been promoted as less harmful substitutes for cigarettes.

## Supplementary Information

**Additional file 1.**

## Data Availability

The datasets generated during and/or analyzed during the current study are available in the Global Tobacco Surveillance System Data, https://nccd.cdc.gov/GTSSDataSurveyResources/Ancillary/DataReports.aspx?CAID=2.
