# Supplementary material for: Association between perceived harm of tobacco and intention to quit: a cross-sectional analysis of the Vietnam Global Adult Tobacco Survey
Source: BMC Public Health. 2022 May 6;22:909. doi: 10.1186/s12889-022-13348-w (PMC9077992; doi:10.1186/s12889-022-13348-w)
Supplement: Supplementary file 1 — Additional file 1. [file 12889_2022_13348_MOESM1_ESM.docx]

Supplemental Table 1: Sensitivity analysis: Factors associated with intention to quit by tobacco user groups (included all observations having missing information on concerned variables)

|  | **Multiple logistic regression model^a^** | | | | |
| --- | --- | --- | --- | --- | --- |
|  | **Cigarette-only users** | **Waterpipe tobacco only-users** | | **Dual users** | |
|  | OR (95%CI) | OR (95%CI) | | OR (95%CI) | |
| **Demographic characteristics** |  |  | |  | |
| Age group |  |  | |  | |
| 18-24 | 1 | 1 | | 1 | |
| 25-44 | 0.56 [0.26-1.19] | 0.70 [0.13-3.62] | | 0.81 [0.09-7.00] | |
| 45-64 | 0.67 [0.30-1.51] | 0.90 [0.16-5.21] | | 0.28 [0.03-3.13] | |
| ≥65 | 0.49 [0.20-1.21] | 1.28 [0.16-10.19] | | _ | |
| Education level |  |  | |  | |
| Primary or less | 1 | 1 | | 1 | |
| Secondary school | 1.14 [0.75-1.73] | 0.87 [0.33-2.28] | | 0.27 [0.04-1.70] | |
| High school or higher | 1.17 [0.71-1.94] | 2.16 [0.71-6.64] | | 0.15* [0.02-0.98] | |
| Marital status |  |  | |  | |
| Unmarried | 1 | 1 | | 1 | |
| Married | 0.83 [0.43-1.61] | 1.25 [0.28-5.60] | | 1.29 [0.17-9.92] | |
| Separated/divorced/widowed | 1.13 [0.48-2.69] | 1.57 [0.19-13.31] | | 4.11 [0.27-62.65] | |
| Having children at home |  |  | |  | |
| No | 1 | 1 | | 1 | |
| Yes | 1.27 [0.86-1.87] | 1.85 [0.93-3.67] | | 1.08 [0.30-3.88] | |
| **Tobacco use behaviors** |  |  | |  | |
| Age at tobacco use initiation | 1.04 [1.00-1.07] | 1.04 [1.00-1.08] | | 1.07 [0.96-1.21] | |
| Number of cigarettes per day | 1.00 [0.97-1.02] | _ | | 0.96 [0.88-1.05] | |
| Number of WP sessions smoked per day | _ | 1.01 [0.97-1.05] | | 1.02 [0.96-1.09] | |
| Time to the first use of cigarette/waterpipe after waking | | |  | |  |
| Within 5 mins | 1 | 1 | | 1 | |
| 6-30 mins | 1.34 [0.84-2.14] | 1.06 [0.55-2.03] | | 1.21 [0.31-4.81] | |
| 31-60 mins | 1.73* [1.00-2.98] | 1.15 [0.48-2.75] | | 0.31 [0.06-1.73] | |
| >60 mins | 2.03* [1.16-3.55] | 1.94 [0.54-6.96] | | 0.87 [0.13-5.72] | |
| **Perceived harm from tobacco use** |  |  | |  | |
| Cigarette and waterpipe use causes severe illness |  |  | |  | |
| Do not cause severe illness | 1 | _ | | _ | |
| Only waterpipe causes severe illness | 10.06* [1.45-69.51] | 0.16* [0.03-0.79] | | _ | |
| Only cigarette causes severe illness | 13.60** [2.26-81.77] | 2.47 [0.53-11.41] | | 0.06* [0.00-0.70] | |
| Both cause severe illness | 8.89** [1.78-44.49] | 1 | | 1 | |
| Perceived harm from their tobacco product compares to another |  |  | |  | |
| Less harmful | 1 | 1 | | _ | |
| Equally harmful | 0.96 [0.63-1.46] | 1.33 [0.55-3.22] | | _ | |
| More harmful | 1.66* [1.03-2.65] | 1.49 [0.23-9.79] | | _ | |
| **Regulation/policy effect** |  |  | |  | |
| Tobacco use regulation at home |  |  | |  | |
| No ban | 1 | 1 | | 1 | |
| Partial ban | 1.13 [0.80-1.59] | 4.68*** [2.17-10.12] | | 1.73 [0.48-6.27] | |
| Comprehensive ban | 1.65 [0.95-2.88] | 3.28* [1.05-10.24] | | 1.01 [0.24-4.30] | |
| Exposed to anti-smoking campaigns or encouragement to quit information (within the last 30 days) |  |  | |  | |
| No | 1 | 1 | | 1 | |
| Yes | 1.61* [1.06-2.45] | 2.07 [0.88-4.87] | | 5.61** [1.60-19.67] | |
| Exposed to advertisements/signage to promote tobacco products (within the last 30 days) |  |  | |  | |
| No | 1 | 1 | | 1 | |
| Yes | 0.96 [0.61-1.49] | 1.01 [0.34-3.01] | | 2.10 [0.47-9.27] | |
| N | 969 | 286 | | 127 | |
| * p<0.05 ** p<0.01 *** p<0.001 | | | | | |
| ^a^ Adjusted for all factors in the model | | | | | |
